# Impaired Oxidative Stress Markers and Activities of Matrix Metalloproteinases in Plasma of Patients with Alzheimer’s Disease, Emphasizing Sex and *APOE* ε4 Allele Possession

**DOI:** 10.3390/ijms26188790

**Published:** 2025-09-09

**Authors:** Dominika Radošinská, Marta Kollárová, Ivana Shawkatová, Vladimíra Ďurmanová, Zuzana Párnická, Juraj Javor, Petra Brandoburová, Štefan Harsányi, Jana Radošinská

**Affiliations:** 1 Institute of Medical Biology, Genetics and Clinical Genetics, Faculty of Medicine, Comenius University in Bratislava, Sasinkova 4, 811 08 Bratislava, Slovakia; dominika.radosinska@fmed.uniba.sk (D.R.); stefan.harsanyi@fmed.uniba.sk (Š.H.); 2 Premedix Academy, 811 02 Bratislava, Slovakia; marta.husseinova@fmed.uniba.sk; 3 Institute of Immunology, Faculty of Medicine, Comenius University in Bratislava, 811 08 Bratislava, Slovakia; ivana.shawkatova@fmed.uniba.sk (I.S.); vladimira.durmanova@fmed.uniba.sk (V.Ď.); zuzana.parnicka@fmed.uniba.sk (Z.P.); juraj.javor@fmed.uniba.sk (J.J.); 4 Centrum MEMORY, 851 03 Bratislava, Slovakia; brandoburova@centrummemory.sk; 5 Department of Psychology, Faculty of Arts, Comenius University in Bratislava, 811 08 Bratislava, Slovakia; 6 Institute of Physiology, Faculty of Medicine, Comenius University in Bratislava, Sasinkova 2, 811 08 Bratislava, Slovakia

**Keywords:** Alzheimer’s disease, matrix metalloproteinases, oxidative stress, sex differences

## Abstract

Alzheimer’s disease (AD) is a progressive neurodegenerative disorder characterized by Aβ accumulation, tau pathology, and associated oxidative and inflammatory changes, including matrix metalloproteinase (MMP) involvement. This study investigated plasma markers of oxidative damage, carbonyl stress, antioxidant status, and activities of MMP-9 and MMP-2 in AD patients and controls. Spectrophotometric and fluorescent assays were used to assess oxidative and carbonyl stress markers, while MMP activities were measured by gelatin zymography. AD patients exhibited significantly increased protein oxidation, carbonyl stress, and GSH/GSSG ratio, along with reduced total antioxidant capacity and superoxide dismutase activity. Plasma MMP-2 activity was elevated in AD patients, whereas MMP-9 activity showed no significant difference. Notable sex-specific patterns were observed: MMP-2 activity was higher in women with AD than in control women, while MMP-9 activity was increased in men with AD compared with control men. Fructosamine levels were elevated in men regardless of AD status and in AD women versus control women. *APOE* ε4 status had no significant effect on oxidative stress markers or MMP-9 activity, though higher MMP-2 activity in non-carriers with AD suggests its potential protective role. These findings support the relevance of peripheral biomarkers in AD and indicate sex-dependent pathways that may guide personalized therapeutic strategies.

## 1. Introduction

Alzheimer’s disease (AD) is a multifactorial, progressive, neurodegenerative disorder characterized by cognitive impairment due to the death of neurons. Typical neuropathological hallmarks of AD include amyloid plaques and neurofibrillary tangles. Amyloid plaques are formed by the proteolytic cleavage of the amyloid precursor protein, which produces amyloid-β (Aβ) peptides that aggregate into insoluble fibrils. Neurofibrillary tangles consist of hyperphosphorylated tau proteins [1,2]. Beyond amyloid and tau pathophysiology, there is growing recognition of the roles played by inflammation, vascular, and infection factors in AD [3]. The vast majority of AD cases are late-onset or sporadic AD without specific genetic mutations, which develop after the age of 65 years. Sporadic AD might be established due to an interplay of multiple genetic risk factors, environmental factors, and life events. The strongest known genetic risk factor for developing sporadic AD is the presence of an apolipoprotein E (*APOE*) ε4 allele [4,5]. The *APOE* ε4 influences AD pathogenesis through multiple mechanisms, including the regulation of Aβ clearance, tau phosphorylation, blood–brain barrier integrity, inflammation, and synaptic maintenance [6]. *APOE* ε4, in particular, alters lipid metabolism, impairs cytoskeletal structure, disrupts glucose and oxidative metabolism, and affects processes such as autophagy, cytokine release, and neuroinflammation [3]. By promoting proinflammatory immune responses, *APOE* ε4 has also been linked to increased oxidative stress, as demonstrated in both animal and human AD studies [7]. While some studies have reported a link between the *APOE* ε4 allele and altered oxidative stress markers [8,9,10], others have found no such association [11,12]. In addition to the *APOE* genotype, women exhibit a higher prevalence and nearly double the incidence compared with men [13]. Although the exact cause of this difference remains unclear, the role of 17-b-estradiol in neuronal and glial plasticity has been recognized. Its postmenopausal decline may contribute to the increased risk of AD in women [14]. Beyond sex hormones, genetic factors, differences in brain structure, and variations in responses to inflammation and psychosocial stress may also underlie the greater intrinsic risk of AD in women compared with men [15]. The main challenges of AD include its development without clear, specific clinical signatures, delayed or complex diagnosis, and limited treatment options that primarily address the consequences rather than the causes. As a result, many research groups are working to identify and study new biomarkers that could aid in prevention, slow disease progression, enable earlier diagnosis, and allow for the use of less invasive biological samples in research [1].

The cellular redox balance is essential for the normal functioning and survival of all cells. Disruption in this balance can lead to oxidative damage and contribute to the development of various human diseases, including metabolic and neurodegenerative disorders [10,16]. This dysregulation is caused by the overproduction of oxidants and the depletion of antioxidants, leading to modifications of lipids, proteins, and nucleic acids. In this context, oxidative stress markers can serve as preliminary indicators of pathological processes. These markers can be measured in various biological fluids, tissues, or organelles. Altered oxidative stress parameters have already been observed in comparisons between AD patients and control groups [17]. Although it remains unclear whether oxidative damage is a cause or consequence of pathological processes in the brain or circulation, and oxidative stress markers are not specific to AD, their use alongside other biomarkers—as part of a panel—may offer deeper insight into an individual’s risk and disease progression. Regarding AD patients, the mechanisms involved in oxidative stress processes are mitochondrial dysfunction, tau hyperphosphorylation, Aβ peptide accumulation, impaired metal homeostasis, inflammation, and lack of antioxidant enzymes [18]. Along with oxidative damage, dysregulation of matrix metalloproteinases (MMPs) has also been implicated in AD [19,20]. MMPs are large, proteolytic, structurally related zinc-dependent enzymes participating in various physiological and pathophysiological processes. They are generally involved in extracellular matrix remodeling, development, morphogenesis, inflammation, and signaling pathways [21]. MMP-9 and MMP-2, two gelatinases, are often associated with AD due to their potential role in its pathogenesis. They are thought to influence neural network remodeling, neuroinflammation, immune responses, blood–brain barrier disruption, Aβ and tau metabolism, as well as neuronal survival and death [22,23]. However, the available data regarding their alterations in AD are inconsistent and, at times, contradictory [20].

Taking the above into account, this study aimed to determine the circulating activities of MMP-9 and MMP-2, along with a panel of oxidative stress markers, in patients with late-onset AD and controls. The goal was to obtain a broader range of biomarkers to gain a comprehensive view of potential changes and explore possible associations. Given that sex is a significant biological and clinical modifier in AD, particular emphasis was placed on investigating sex-related differences. Additionally, the relations between MMPs, oxidative stress, and antioxidant status—encompassing both enzymatic and non-enzymatic molecules—were analyzed in the context of *APOE* ε4 carrier status.

## 2. Results

### 2.1. Study Population

The study group consisted of 104 patients diagnosed with sporadic AD. Cognitive status was assessed using the Montreal Cognitive Assessment (MoCA), with a mean score below the standard cut-off of 26. Only ICD-10 diagnoses F00.1 and F00.2 were included, corresponding to forms of dementia related to AD. Diagnosis F00.1 refers to late-onset AD, defined as onset after 65 years of age, while F00.2 represents the mixed form of AD, where dementia results from a combination of AD pathology and other factors such as vascular dementia. Notably, no cases of pure vascular dementia were included in the study. The reference group in this case–control study comprised 73 unrelated, cognitively normal individuals. None had a family history of AD or other forms of dementia. Table 1 summarizes the key characteristics of the study groups.

### 2.2. Distribution of the APOE Gene Alleles in Study Participants

Table 2 summarizes *APOE* allele distribution within the study participants. A strong association was found between the possession of the *APOE* ε4 allele and AD cases (*p* < 0.0001).

### 2.3. Alteration of Oxidative Stress Markers and Antioxidant Status in the Plasma of AD Patients

As shown in Table 3, the analysis of oxidative and carbonyl stress parameters in plasma revealed several group differences. Levels of advanced oxidation protein products (AOPP), which reflect oxidative damage to proteins, were significantly elevated in the AD group compared with controls (*p* = 0.001). Both markers of carbonyl stress—fructosamine, indicating early protein glycation (*p* = 0.0011), and advanced glycation end products (AGEs), representing advanced glycation (*p* = 0.0283)—were significantly increased in AD patients. Given the elevated carbonyl stress markers in the AD group along with a higher prevalence of diabetes mellitus, potential correlations between the presence of diabetes and levels of fructosamine or AGEs within the AD group were also examined. However, the analyses showed no significant associations between diabetes and either fructosamine (r = 0.1241, *p* = 0.2232) or AGEs (r = −0.0329, *p* = 0.7456). Additionally, the ratio of reduced to oxidized glutathione (GSH/GSSG ratio), a general indicator of oxidative stress, was significantly higher in the AD group compared with control individuals (*p* = 0.0012).

Data on total antioxidant capacity (TAC), superoxide dismutase (SOD) activity, ferric reducing antioxidant power (FRAP) of plasma, and catalase (CAT) activity are presented in Table 4.

Due to the significant age difference between the experimental groups, we first applied correlation analyses to assess whether oxidative stress parameters were associated with participants’ age. Next, we performed multiple regression analyses to examine whether age and disease status independently influenced the parameters that showed significant correlations. The regression results indicated that AOPP levels were not significantly affected by either age or disease, while fructosamine levels were influenced by disease status. A significant interaction between age and disease status suggests that both fructosamine and TAC parameters are influenced differently in individuals with AD compared with controls (Table 5). For actual data and their variation, see Supplemental Figure S1.

### 2.4. Elevated MMP-2 Activity, but Not MMP-9, in the Plasma of AD Patients

**Figure 1:**
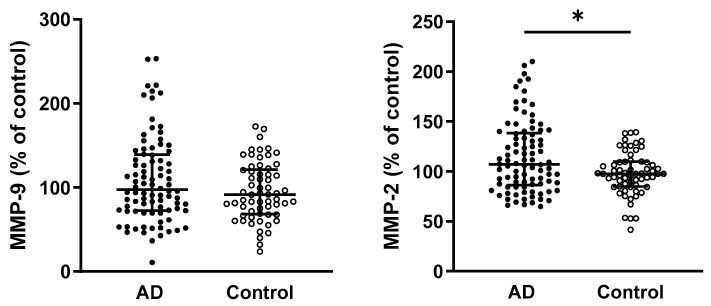
Comparison of circulating MMP-9 and MMP-2 activity in AD patients and controls. Statistical significance: * represents *p* < 0.05. Data are presented as the medians and interquartile ranges (Q1; Q3). Differences between study groups were analyzed using a Mann–Whitney test. Abbreviations: AD—Alzheimer’s disease, MMP—matrix metalloproteinase.

In the analysis of MMP-9 activity, no significant differences were observed between AD patients and controls (*p* = 0.2134). In contrast, MMP-2 activity was significantly higher in AD patients compared with the control group (*p* = 0.025) (Figure 1).

A multivariable regression analysis was performed to account for the potential influence of age on the significant findings. The results revealed that MMP-2 activity was significantly associated with the disease status (*p* = 0.0452) and with the interaction between age and disease (*p* = 0.0332).

### 2.5. Sex Differences in Oxidative Stress Parameters and MMPs Activities

**Figure 2:**
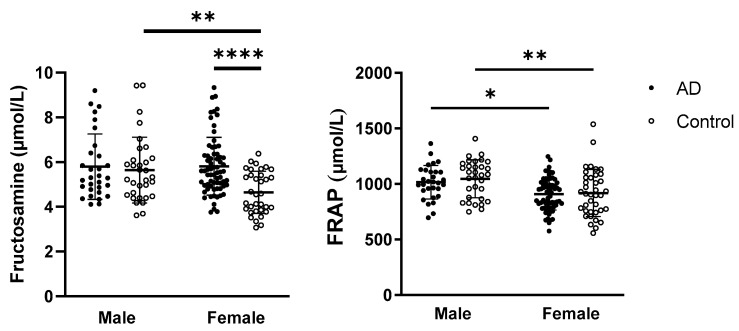
Fructosamine and FRAP levels in relation to sex and disease status in AD patients and control subjects. Statistical significance: * represents *p* < 0.05, ** *p* < 0.01, **** *p* < 0.0001. Data are presented as the mean ± standard deviation. The impact of disease status and sex was evaluated within each group using a two-way ANOVA followed by Tukey’s multiple comparisons post hoc test. Abbreviations: AD—Alzheimer’s disease, FRAP—ferric reducing antioxidant power.

A two-way ANOVA revealed statistically significant effects of both sex (*p* = 0.0163; F_(1,160)_ = 5.900) and disease status (*p* = 0.0007; F_(1,160)_ = 11.86) on the early glycation marker fructosamine. Additionally, a significant interaction between sex and disease status was observed (*p* = 0.0081; F_(1,160)_ = 7.161). For the FRAP parameter, a significant effect of sex was observed (*p* < 0.0001; F_(1,163)_ = 18.60). Data with statistically significant results from multiple comparisons are presented in Figure 2.

Other oxidative stress parameters—including levels of thiobarbituric acid reactive substances (TBARS), AOPP, AGEs, GSH/GSSG, and TAC, as well as the activities of SOD and CAT—did not show any association between sex and disease status.

**Figure 3:**
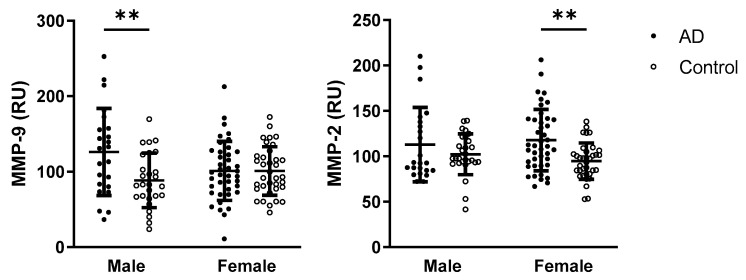
Circulating MMP activities in relation to sex and disease in AD patients and controls. Statistical significance: ** represents *p* < 0.01. Data are presented as the mean ± standard deviation. The impact of disease status and sex was evaluated within each group using a two-way ANOVA followed by Tukey’s multiple comparisons post hoc test. Abbreviations: AD—Alzheimer’s disease, MMP—matrix metalloproteinases, RU—relative units.

When assessing the effect of sex and disease on MMP-9 activity, the two-way ANOVA test revealed a significant interaction between disease and sex (*p* = 0.0134; F_(1,121)_ = 6.293), as well as a significant effect of disease status (*p* = 0.0125; F_(1,121)_ = 6.427). MMP-9 activity was significantly higher in male AD patients compared with male controls. Similarly, for MMP-2 activity, a significant interaction between sex and disease was observed (*p* = 0.0477; F_(1,122)_ = 4.001), and disease alone was also identified as a significant factor (*p* = 0.0206; F_(1,122)_ = 5.504). In contrast to MMP-9, MMP-2 activity was significantly higher in female AD patients compared with female controls. Data with statistically significant results from multiple comparisons are presented in Figure 3.

### 2.6. Emphasis on the Possession of APOE ε4 on Parameters of Oxidative Stress and Activities of Matrix Metalloproteinases

**Figure 4:**
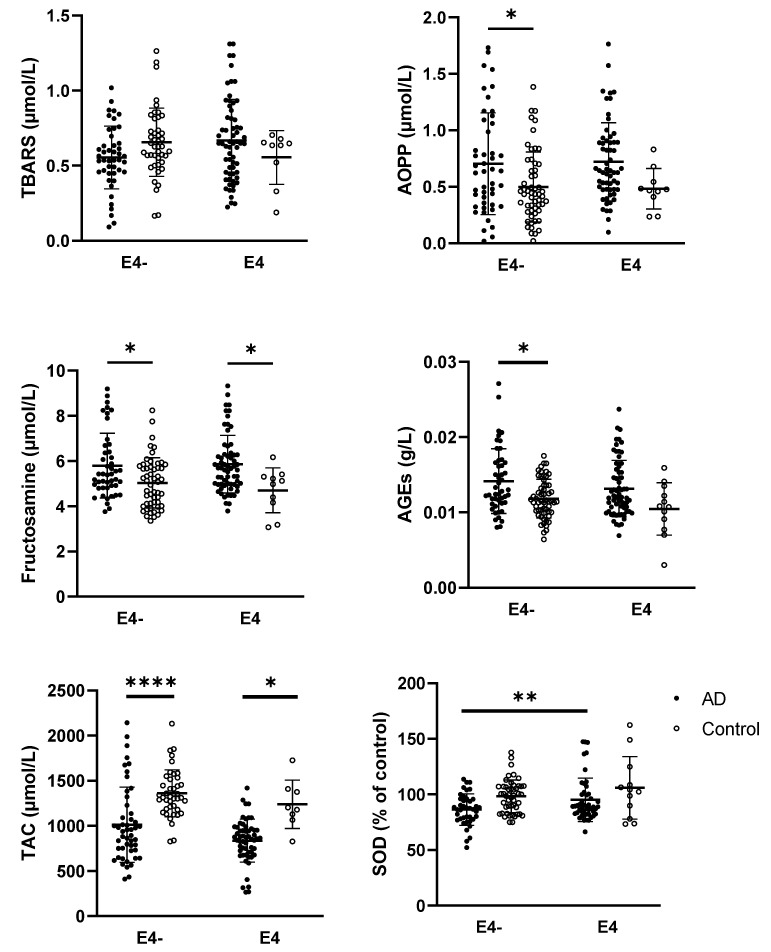
Parameters of oxidative stress in relation to the *APOE* ε4 allele and disease. Statistical significance: * represents *p* < 0.05, ** *p* < 0.01, **** *p* < 0.0001. Data are presented as the mean ± standard deviation. The impact of disease and *APOE* status was evaluated within each group using a two-way ANOVA followed by Tukey’s multiple comparisons post hoc test. Abbreviations: AD—Alzheimer’s disease, TBARS—thiobarbituric acid reactive substances, AOPP—advanced oxidation protein products, AGEs—advanced glycation end products, GSH/GSSG—the reduced and oxidized glutathione ratio, TAC—total antioxidant capacity, SOD—superoxide dismutase, E4−—individuals without the possession of *APOE* ε4 allele, E4—individuals with the possession of *APOE* ε4 allele.

A two-way ANOVA revealed a significant interaction between the *APOE* ε4 allele and AD status for the TBARS parameter (*p* = 0.0324; F_(1,157)_ = 4.658). In contrast, other parameters—including fructosamine (*p* < 0.0001; F_(1,168)_ = 17.19), AGEs (*p* = 0.0016; F_(1,175)_ = 10.23), AOPP (*p* = 0.0015; F_(1,164)_ = 10.36), GSH/GSSG ratio (*p* = 0.0130; F_(1,159)_ = 6.314), TAC (*p* < 0.0001; F_(1,146)_ = 32.75) and SOD (*p* < 0.0001; F_(1,141)_ = 16.22)—showed a significant effect of disease status only. For detailed data and statistically significant multiple comparisons, refer to Figure 4.

**Figure 5:**
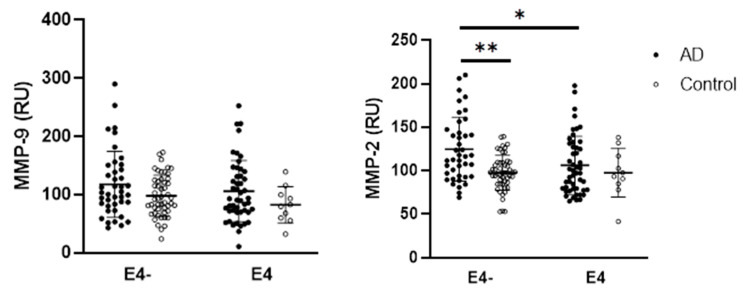
Activities of MMPs in relation to the *APOE* ε4 allele and disease. Statistical significance: * represents *p* < 0.05, ** *p* < 0.01. Data are presented as the mean ± standard deviation. The impact of disease and *APOE* status was evaluated within each group using a two-way ANOVA followed by Tukey’s multiple comparisons post hoc test. Abbreviations: AD—Alzheimer’s disease, MMP—matrix metalloproteinases, E4−—individuals without the possession of *APOE* ε4 allele, E4—individuals with the possession of *APOE* ε4 allele, RU—relative units.

Furthermore, when analyzing the impact of *APOE* ε4 allele status and disease on MMP activities, a significant effect of disease was observed for MMP-2 activity (*p* = 0.0424; F_(1,142)_ = 4.192). However, neither *APOE* ε4 allele status nor disease had a significant effect on MMP-9 activity. For detailed data and statistically significant multiple comparisons, refer to Figure 5.

## 3. Discussion

Available data suggest a close relationship between amyloid-induced MMP activation and oxidative stress [19,24], although findings are not always consistent and are sometimes even contradictory [20]. Therefore, this study aimed to analyze a broader panel of oxidative stress markers, along with the activities of MMP-2 and MMP-9 in the plasma of AD patients and control subjects.

Regarding the basic characterization of the study groups, MoCA scores—used for cognitive screening [25]—were, as expected, significantly lower in AD patients compared with controls. In addition, among the various comorbidities commonly associated with AD, our study found a significant difference only in the prevalence of diabetes mellitus. The individuals with diabetes had type 2 diabetes mellitus and were treated with oral antidiabetic medications. It is well established that diabetes and AD share several pathological features, including impaired insulin signaling, dyslipidemia, decreased levels of choline acetyltransferase, increased production of AGEs and inflammatory mediators, as well as the accumulation of Aβ peptides and tau proteins [26]. These mechanisms contribute to the cognitive decline and neurodegeneration characteristic of Alzheimer’s dementia. For this reason, AD is sometimes referred to as “type 3 diabetes” [27,28]. Although a statistically significant age difference existed between the groups, multivariable regression analysis confirmed that age did not influence the observed differences between AD patients and controls.

Oxidative stress is a contributing factor in a wide range of chronic diseases, including neurodegenerative disorders such as AD and metabolic disorders like diabetes mellitus. Disruption of redox homeostasis can drive disease progression and worsen clinical outcomes. Various underlying mechanisms have been identified; for a detailed review, see [29]. In this study, we first analyzed markers of oxidative damage in plasma and compared their levels between AD patients and control subjects. Levels of TBARS—a marker of lipid peroxidation—did not differ significantly in our study population. This finding contrasts with several previous studies reporting elevated TBARS levels in AD patients [19,30,31]. However, the literature on TBARS remains inconsistent and somewhat controversial [16]. This variability may be attributed to differences in assay sensitivity or specificity, experimental protocols, or genetic factors such as the *APOE* genotype [32], which was also found to be potentially relevant in this study. In contrast, we observed increased levels of AOPP—a marker of protein oxidation—which aligns with a previous report [11]. Beyond oxidative damage, proteins can also be modified by glycation, a non-enzymatic reaction in which sugars bind to free amino acid groups on proteins [33]. Fructosamine, an early glycation marker, showed significant increases in the AD group. Along with AGEs, these findings are consistent with prior studies [34,35]. Both oxidized and glycated proteins could potentially contribute to metabolic dysfunction through protein misfolding and accumulation, thereby playing a role in AD progression [8,36]. AGEs are also implicated in type 2 diabetes mellitus, ageing, and AD pathogenesis [37], while their accumulation contributes to neuronal dysfunction and death [38]. In this study, we focused on fluorescent AGEs, which are primarily composed of pentosidine [39]. Pentosidine is a potent cross-linker involved in the formation of fibrillar structures in the neuropil and amyloid plaques [40]. Therefore, it is plausible to hypothesize that specific AGEs could serve as early biomarkers for AD detection [41,42,43]. Notably, it should be emphasized that despite the higher prevalence of diabetes in the AD group, multivariate regression analyses showed that the increase in carbonyl stress markers—both fructosamine concentration and AGE levels—was not dependent on the presence of diabetes in this study.

Oxidative stress generally results in a decline in both enzymatic and non-enzymatic antioxidant defense mechanisms. Consistent with previous studies [9,12,44], we observed decreased SOD enzyme activity and reduced TAC levels in plasma samples from AD patients, although the CAT enzyme activity and the levels of the FRAP marker did not show significant differences. It was proposed that alterations in antioxidant enzyme activities may stem from imbalances in metal cofactor concentrations in patients with AD [45]. Furthermore, excessive free radical production, as indicated by elevated oxidative stress markers in this study, can deplete plasma antioxidant defenses. Overall, our findings suggest an impaired antioxidant defense system that fails to adequately compensate for increased oxidative stress, potentially contributing to the neuroinflammatory processes characteristic of AD. Dietary antioxidants can help restore this balance, thereby reducing the risk or delaying the progression of AD and related cognitive decline [12]. However, our data also showed a higher GSH/GSSG ratio in AD patients compared with controls, suggesting a preserved thiol-disulphide redox balance. Several factors may account for this unexpected finding, including disease severity, methodological differences, medication effects, or compensatory mechanisms [46]. Glutathione (GSH) serves as a substrate for glutathione peroxidase, and reduced glutathione peroxidase activity—commonly reported in AD patients—can lead to elevated GSH levels [12,47,48].

Given that females are more susceptible to AD, this study also focused on sex-specific differences. Regarding oxidative stress markers, fructosamine and FRAP levels showed sex-dependent variations, while female controls had lower fructosamine levels compared to male controls, and both female groups—AD and control—exhibited lower FRAP levels than males. Sex differences in FRAP level, with higher values in men than in women, were already noted in young healthy subjects [49,50], and middle-aged people [51], although the difference was not always statistically significant [52]. Sex differences in fructosamine levels, with higher values in men than in women, were noted in this study only in control participants, which is consistent with previous observations [53]. In AD patients, who exhibited higher levels of fructosamine, sex-related changes were suppressed.

To further explore oxidative stress and inflammation-related markers in AD, recent studies have identified specific plasma MMPs as potential biomarkers for both preclinical and clinical stages, as well as for evaluating disease severity. Among these, the gelatinases MMP-9 and MMP-2 are particularly notable for their capacity to cleave the Aβ peptides and facilitate their clearance, as demonstrated in vitro and in transgenic models of AD [20,22,54,55,56]. Moreover, MMP-2 has been shown to be upregulated during the early and intermediate stages of AD pathogenesis, while its incapacity to cleave hyperphosphorylated tau may contribute to the accumulation of tau aggregates [57]. Notably, MMP-2 is constitutively expressed, whereas MMP-9 is largely inducible. This difference is partly due to the presence of proinflammatory transcription factor binding sites in the MMP-9 gene—features that are absent in the MMP-2 gene. Interestingly, higher plasma MMP-9 concentration has been shown to be associated with a greater risk of AD development in *APOE* ε4 carriers with mild cognitive impairment [58]. In this study, differential patterns were observed between the two gelatinases: while plasma MMP-9 activity did not differ significantly between AD patients and controls, MMP-2 activity was significantly elevated in AD patients. Despite increasing interest in this area, findings on plasma (or cerebrospinal fluid) MMP activities or MMP levels in AD remain inconsistent [20]. To elucidate the source of possible discrepancies, we further investigated whether MMP activity was associated with sex using multivariate analyses. Although no overall sex-based differences in the activity of both MMPs were observed, subgroup analyses revealed that AD females exhibited increased MMP-2 activity compared to female controls, whereas AD males showed higher MMP-9 activity than male controls. It has been proposed that biological sex significantly modifies the effect of MMP-9 on AD etiopathogenesis, with MMP-9 concentration showing stronger clinical relevance in women [59]. However, it is not yet known whether this observation also extends to MMP-9 activity. Another study focused on a range of MMPs and their endogenous inhibitors observed that male sex was significantly associated with increased levels of AD biomarkers [60]. Observed sex differences could be influenced by several factors, including distinct, sex-specific AD candidate genes [13], sex-specific transcriptomic signature [61], sex steroid hormones [14], age-related hormonal changes, brain structural differences, inflammation, microglial activity [62], and others—for reviews, see [15,63].

In addition to biological sex, *APOE* ε4 status may also contribute to the inconsistencies regarding the MMPs and AD observed across databases. From this perspective, MMP-9 appears to be less affected. One possible explanation is the greater inter-individual variability in MMP-9 activity, which may be influenced not only by AD pathophysiology but also by other age-related comorbidities, such as cardiovascular diseases. In contrast, MMP-2 activity, which was found to be elevated in AD patients in this study, was higher in *APOE* ε4 AD non-carriers compared with *APOE* ε4 AD carriers. This observation raises the possibility that increased MMP-2 activity may represent a protective rather than a detrimental response. Notably, *APOE* ε4 allele status did not significantly influence any of the oxidative stress markers measured in this study.

A relevant question is whether oxidative stress markers and gelatinase activity could aid in the differential diagnosis of dementia. In addition to AD, common types include vascular dementia (the second most prevalent), Lewy body dementia, and frontotemporal dementia [64]. Oxidative stress contributes to the pathogenesis of both AD and vascular dementia: in AD, amyloid-β accumulation triggers reactive oxygen species production and neuronal damage, while in vascular dementia, elevated reactive oxygen species, endothelial dysfunction, and reduced nitric oxide are implicated [65]. Oxidative stress also underlies key vascular dementia risk factors such as diabetes, hypercholesterolemia, and hyperhomocysteinemia [66].

Regarding gelatinases, elevated MMP-9—an inflammation-related marker—has been associated with vascular cognitive impairment [67]. However, other studies report higher MMP-9 levels in AD compared with other dementia types [68], highlighting inconsistencies in the findings. Therefore, while promising, the diagnostic utility of these markers remains uncertain. It has also been suggested that a single marker may be insufficient for diagnostic accuracy, and that a multimarker panel involving MMPs could be more informative [69].

## 4. Materials and Methods

### 4.1. Study Groups/Participants

AD diagnosis was determined based on the clinical picture supported by radiologic and cerebrospinal fluid biomarkers following the DSM-IV and NINCDS-ADRDA criteria, as well as the new lexicon for AD [70,71]. All the patients in the study underwent magnetic resonance imaging of the brain, whereas only patients with a Scheltens score of two and more hippocampal atrophy were included in the study. All participants, both patients and controls, were Caucasians of Slovak descent. The AD patients were recruited randomly from various medical centers in Bratislava, including the Psychiatry Clinic FMCU & UHB, 2nd Neurology Clinic FMCU & UHB, and Care Center Centrum MEMORY. Informed consent was obtained from all AD patients or their legally authorized representatives, as well as from the controls participating in the study.

All procedures were conducted in compliance with the International Ethical Guidelines and the Declaration of Helsinki. The study received approval from the Ethical Committee of the University Hospital Bratislava and the Faculty of Medicine, Comenius University in Bratislava, Slovak Republic, and the Independent Ethical Committee of the Bratislava Municipality under the No. 05440/2021/HF.

### 4.2. Blood Collection

Blood samples from both study groups were collected into 5–7 mL EDTA-containing vacutainer tubes, primarily in the morning and under fasting conditions. Plasma was separated by centrifugation at 1150× *g* for 10 min at 4 °C and promptly stored at −80 °C for subsequent analyses.

### 4.3. APOE Genotyping

*APOE* genotyping was performed by direct sequencing, targeting two single-nucleotide polymorphisms located in the fourth exon of the *APOE* gene, namely rs429358: T > C and rs7412: C > T. The presence of the minor allele at rs429358 typically indicates the ε4 allele, while the minor allele at rs7412 corresponds to the ε2 allele. The combination of rs429358 T and rs7412 C defines the most common ε3 allele. 

Briefly, PCR amplification was carried out using genomic DNA extracted from EDTA-treated blood samples. The reaction utilized a denatured flanking forward primer 5′-ACTGACCCCGGTGGCGGAGGAGACGCGGGC-3′ and a reverse primer 5′-TGTTCCACCAGGGGCCCCAGGCGCTCGCGG-3′. PCR conditions included initial denaturation at 95 °C for 5 min, followed by 40 cycles of denaturation at 95 °C for 1 min, annealing at 68 °C for 30 s, and extension at 72 °C for 30 s, with a final extension step at 72 °C for 7 min, as previously described by Durmanova et al. [72]. The resulting 318 bp PCR products were sequenced using both primers and the BigDye^®^ Terminator v3.1 Cycle Sequencing Kit (Thermo Fisher Scientific, Waltham, MA, USA) on an Applied Biosystems 3130xl Genetic Analyzer (Life Technologies, Carlsbad, CA, USA). Sequence data were analyzed using FinchTV Version 1.4.0 (Geospiza, Inc., Washington, DC, USA), enabling unambiguous determination of the *APOE* genotypes.

### 4.4. Parameters of Oxidative Stress and Antioxidant Status

Markers of oxidative stress, carbonyl stress, and antioxidant status were evaluated in plasma samples following established protocols [73]. For all analyses, the reagents from Merck were purchased.

Briefly, to assess lipid peroxidation, TBARS was measured by mixing 20 µL of undiluted samples and standards (1,1,3,3-tetraethoxypropane, range: 0–10 µmol/L) with 30 µL of deionized water, 20 µL of 0.67% thiobarbituric acid, and 20 µL of glacial acetic acid. The mixture was incubated at 95 °C for 45 min, cooled to room temperature, and then centrifuged. The upper phase was transferred to a new microplate, and fluorescence was measured at λ_ex_ = 515 nm and λ_em_ = 553 nm.

To assess protein oxidative damage, AOPPs were determined by mixing 200 µL of 5× diluted samples and standards (mixture of chloramine T and potassium iodide, range: 0–100 µmol/L) with 20 µL of glacial acetic acid and measuring absorbance at λ = 340 nm after 2 min of incubation.

For carbonyl stress analysis, AGEs were measured. Samples were mixed with PBS, and autofluorescence was measured at λ_ex_ = 370 nm and λ_em_ = 440 nm. After adding standards, fluorescence was measured again at the same wavelengths. Fructosamine was measured by mixing samples and standards with a solution containing sodium carbonate buffer and nitro blue tetrazolium, followed by incubation and measurement of absorbance at λ = 530 nm.

The thiol-disulfide redox balance, represented by the ratio of reduced and oxidized glutathione (GSH/GSSG), was examined as well. For GSH, 10 µL of 40× diluted samples and standards (1 mmol/L L-glutathione reduced) were mixed with 10 µL of O-phthalaldehyde (1 mg/mL) and 180 µL of PBS (100 mmol/L with 2.5 mM EDTA-Na_2_). After incubation (15 min. at room temperature), the fluorescence of the mixture was measured at λ_ex_ = 350 nm and λ_em_ = 460 nm. For GSSG, 25 µL of 40× diluted samples and standards ((-)-glutathione, oxidized) were mixed with 10 µL of N-ethylmaleimide (5 mg/mL). The mixture was incubated for 40 min. at room temperature. Then, 10 µL of the mixture was pipetted into a new dark microplate with the addition of 10 µL O-phthalaldehyde (1 mg/mL) and 180 µL NaOH (0.1 mmol/L). After incubation (15 min., gentle vortexing at room temperature in the dark), fluorescence was measured λ_ex_ = 350 nm and λ_em_ = 460 nm.

FRAP was assessed as a marker of antioxidant power. First, a fresh, warmed (37 °C) FRAP reagent was prepared (containing 3 mol/L acetate buffer, pH 3.6; 10 mmol/L tripyridyl-s-triazine; 20 mmol/L FeCl_3_ × 6H_2_O and deionized water), and 200 µL of this reagent was pipetted into the microplate; the absorbance was measured at λ = 593 nm as a blank. Subsequently, 20 µL of 5× diluted samples and standards (0–1000 µmol/L FeSO_4_*7H_2_O) was added and gently vortexed. After 4 min, the absorbance was measured at λ = 593 nm again.

To determine the antioxidant status in plasma, TAC was evaluated. 20 µL of 5× diluted samples and standards (1 mmol/L TROLOX) were mixed with 200 µL of acetate solution (containing 0.4 mol/L CH_3_COONa reagent and 0.4 mol/L glacial acetic acid, pH 5.8). After the measurement of the absorbance (at 660 nm) as a blank, the ABTS solution (20 µL) was added. Then, the mixture was incubated for 5 min by gentle vortexing and re-measured under the same conditions.

Additionally, the activities of SOD and CAT, enzymatic markers of antioxidant defense, were assessed in plasma samples of AD patients and controls. SOD activities were measured using a commercial kit (Arbor assay, cat. number: K028-H1, Ann Arbor, MI, USA), while CAT activities were determined according to Aebi et al. (1984) [74]. The activities of both SOD and CAT were recalculated as percentages of the control values.

### 4.5. Activities of MMPs

To assess the activity of circulating MMPs, specifically MMP-2 and MMP-9, gelatin zymography was employed, following established procedures [73]. In brief, plasma samples were loaded onto 10% polyacrylamide gels containing gelatin (2 mg/mL) as a substrate. Electrophoresis was conducted under denaturing, but not reducing, conditions. Subsequently, the gels underwent two 20 min washes in 50 mM Tris-HCl (pH 7.4, containing 2.5% Triton X-100), followed by overnight incubation at 37 °C in a “refolding” buffer containing Ca^2+^ ions. The gels were then stained with 1% Coomassie Brilliant Blue G-250 for 3 h and destained using a solution of 40% methanol and 10% acetic acid. The zones of lysis, represented by clear bands against a blue background, were quantified via densitometry using ImageJ 1.53k analysis software (NIH, Bethesda, MD, USA).

### 4.6. Statistical Analysis

The data were analyzed, and graphs were generated using GraphPad Prism 9 (GraphPad Software, Inc., San Diego, CA, USA). Outliers were identified and removed using the Grubbs test. The normality of the data was assessed using the D’Agostino-Pearson omnibus test. The difference between study groups was analyzed using an unpaired *t*-test for normally distributed data or a Mann–Whitney test for non-parametric data. Results are presented as means ± standard deviations for normally distributed data (shown as column charts), or as medians with interquartile ranges for non-parametric data (shown as box plots). The chi-square test was used to examine categorical data, such as *APOE* allele distribution in the study groups and the possession of the *APOE* ε4 allele.

Pearson or Spearman correlation analyses were utilized to explore relationships between MMPs/oxidative stress markers and age, and the association between diabetes patients and carbonyl markers. The impact of disease state and *APOE* status or sex was evaluated within each group using a two-way ANOVA (disease × *APOE* genotype/sex), followed by Tukey’s multiple comparisons post hoc tests. For non-parametric data, logarithmically transformed data were utilized to achieve the normal distribution required for ANOVA analysis.

Using linear regression models, we examined the association between age and our measured significant parameters in the whole cohort and in each studied group.

Statistical significance was defined as *p* < 0.05.

## 5. Conclusions

By analyzing a broader panel of oxidative stress markers, this study found increased protein oxidation, carbonyl stress, and GSH/GSSG ratio, along with reduced total antioxidant capacity and SOD activity in the plasma of AD patients compared with controls. With respect to biological sex differences, fructosamine levels were elevated in men irrespective of AD status, and in women with AD compared with control women.

The two gelatinases exhibited distinct activity patterns: plasma MMP-9 activity showed no significant difference between AD patients and controls, whereas MMP-2 activity was significantly elevated in the AD group. This study also highlighted notable sex-related differences: MMP-2 activity was elevated in women with AD compared with control women, whereas MMP-9 activity was higher in men with AD than in control men. These findings support suggestions that in the progression of AD, different sex-dependent pathways are involved, with potential implications for sex-specific therapeutic strategies.

*APOE* ε4 carrier status did not appear to affect the oxidative stress markers or MMP-9 activity observed in this study. However, the higher plasma MMP-2 activity in *APOE* ε4 non-carriers with AD compared with carriers suggests a potential protective role of MMP-2 in AD pathophysiology.

## Figures and Tables

**Table 1:** Basic characteristics of the study population.

	AD Patients (*n* = 104)	Controls (*n* = 73)	*p*-Value
Age (years), mean ± SD	78.9 ± 5.9	69.6 ± 6.6	**<0.0001**
Age at onset, mean ± SD	76.55 ± 6.39	N/A	N/A
Sex (male/female), n	32/72	32/41	0.0825
MoCA score, mean ± SD	16.59 ± 5.8	27.5 ± 1.6	**<0.0001**
Hypertension, %	73.07	63.01	0.1873
Type 2 diabetes mellitus, %	26	12.3	**0.0361**
Tobacco smoker, %	16.3	24.6	0.1845

Abbreviations: AD—Alzheimer’s disease, SD—standard deviation, MoCA—Montreal Cognitive Assessment, N/A—not applicable. Differences between study groups were analyzed using an unpaired *t*-test for continuous variables and Chi-square test for categorical data. *p*-values in bold indicate statistically significant differences.

**Table 2:** Distribution of genotypes and alleles of the *APOE* gene in the study groups.

Genotypes	AD Patients (*n* = 104), *n* (%)	Controls (*n* = 72), *n* (%)	*p*-Value	χ^2^ Test
ε2/ε2	0	1 (1)		
ε2/ε3	8 (8)	11 (15)		
ε2/ε4	4 (4)	2 (3)		
ε3/ε3	41 (39)	47 (65)		
ε3/ε4	39 (38)	11 (15)		
ε4/ε4	12 (11)	0		
ε4+/ε4− carriers	55/49	13/59	**<0.0001**	21.77, 1
Alleles			**<0.0001**	26.72, 2
ε2	12	15		
ε3	129	116		
ε4	67	13		

Abbreviations: AD—Alzheimer’s disease, *APOE*—apolipoprotein E. The Chi-square test was employed for statistical analysis. *p*-values in bold indicate statistically significant differences.

**Table 3:** Parameters of oxidative stress in the plasma of the study participants.

	AD Patients (*n* = 98–104)	Controls (*n* = 69–73)
TBARS (µmol/L)	0.61 ± 0.25	0.65 ± 0.22
AOPP (µmol/L)	0.631 (0.423; 0.935) **	0.460 (0.311; 0.679)
Fructosamine (µmol/L)	5.431 (4.871; 6.276) **	5.123 (4.123; 5.761)
AGEs (g/L)	0.0123 (0.010; 0.015) *	0.0114 (0.010; 0.014)
GSH/GSSG ratio	0.406 (0.325; 0.516) **	0.360 (0.302; 0.411)

Statistical significance: * represents *p* < 0.05, ** *p* < 0.01 compared with controls. Data are presented as the means ± standard deviations for normally distributed data, or medians and interquartile ranges (Q1; Q3) for non-parametric data. Differences between study groups were analyzed using an unpaired *t*-test for normally distributed data, or a Mann–Whitney test for non-parametric data. Abbreviations: AD—Alzheimer’s disease, TBARS—thiobarbituric acid reactive substances, AOPP—advanced oxidation protein products, AGEs—advanced glycation end products, GSH/GSSG—the ratio of reduced to oxidized glutathione.

**Table 4:** Parameters of antioxidant state in the plasma of the study population.

	AD Patients (*n* = 74–104)	Controls (*n* = 55–73)
FRAP (µmol/L)	942.6 ± 158.3	979.2 ± 199.2
TAC (µmol/L)	878.6 (701.5; 1032) ****	1290 (1139; 1447)
CAT activity (% of control)	94.11 ± 40.66	100 ± 30.48
SOD activity (% of control)	88.72 (80.8; 99.09) **	100 (82.82; 107.7)

Statistical significance: ** represents *p* < 0.01, **** *p* < 0.0001 compared with controls. Data are presented as the means ± standard deviations for normally distributed data, or medians and interquartile ranges (Q1; Q3) for non-parametric data. Differences between study groups were analyzed using an unpaired *t*-test for normally distributed data, or a Mann–Whitney test for non-parametric data. Abbreviations: AD—Alzheimer’s disease, FRAP—ferric reducing antioxidant power, TAC—total antioxidant capacity, CAT—catalase, SOD—superoxide dismutase.

**Table 5:** The influence of age and disease on AOPP, fructosamine, and TAC parameters.

	Coefficient	Std. Error	*p*-Value	95% CI
AOPP				
Age	−0.00291	0.00172	0.09277	−0.00631, 0.00049
Disease	−0.15861	0.17065	0.35403	−0.49559, 0.17837
AxD	0.00157	0.0023	0.49541	−0.00297, 0.00611
Fructosamine				
Age	2.46 × 10^−5^	2.14 × 10^−5^	0.25123	−1.8 × 10^−5^, 6.69 × 10^−5^
Disease	0.00483	0.00217	**0.02723**	0.00055, 0.00911
AxD	−6.2 × 10^−5^	2.92 × 10^−5^	**0.03458**	−0.00012, −4.6 × 10^−6^
TAC				
Age	8.92711	7.72268	0.21871	−5.3556, 23.2098
Disease	1084.15	700.389	0.12381	−300.065, 2468.36
AxD	−20.0049	−2.10814	**0.03673**	−38.7587, −1.25062

Abbreviations: AOPP—advanced oxidation protein products, TAC—total antioxidant capacity, AxD—interaction between age and disease status, CI—confidence interval. Multiple regression analyses were performed to examine whether age and disease status influenced the parameters. *p*-values in bold indicate statistically significant differences.

## Data Availability

The dataset is available on request from the authors.

## Supplementary Materials

The following supporting information can be downloaded at: https://www.mdpi.com/article/10.3390/ijms26188790/s1.

## Abbreviations

The following abbreviations are used in this manuscript:

AD	Alzheimer’s disease
Aβ	Amyloid β
MMPs	Matrix metalloproteinases
APOE	Apolipoprotein E
MoCA	Montreal Cognitive Assessment
TBARS	Thiobarbituric acid reactive substances
AOPP	Advanced oxidation protein products
AGEs	Advanced glycation end products
GSH	Reduced glutathione
GSSG	Oxidized glutathione
TAC	Total antioxidant capacity
FRAP	Ferric reducing antioxidant power
SOD	Superoxide dismutase
CAT	Catalase
RU	Relative units
