# Supplementary material for: Impaired Oxidative Stress Markers and Activities of Matrix Metalloproteinases in Plasma of Patients with Alzheimer’s Disease, Emphasizing Sex and APOE ε4 Allele Possession
Source: Int J Mol Sci. 2025 Sep 9;26(18):8790. doi: 10.3390/ijms26188790 (PMC12470211; doi:10.3390/ijms26188790)
Supplement: Supplementary file 1 [file ijms-26-08790-s001.zip › ijms-3759650-supplementary.pdf]

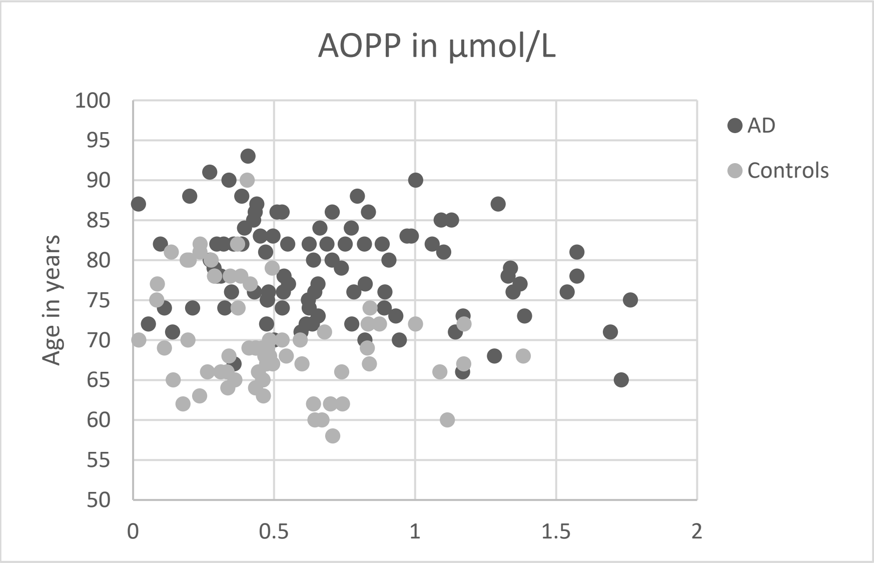

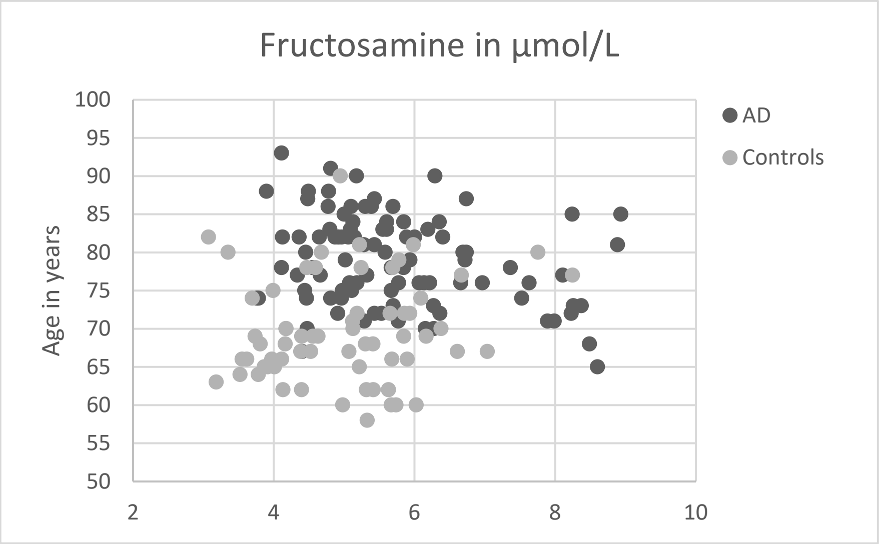

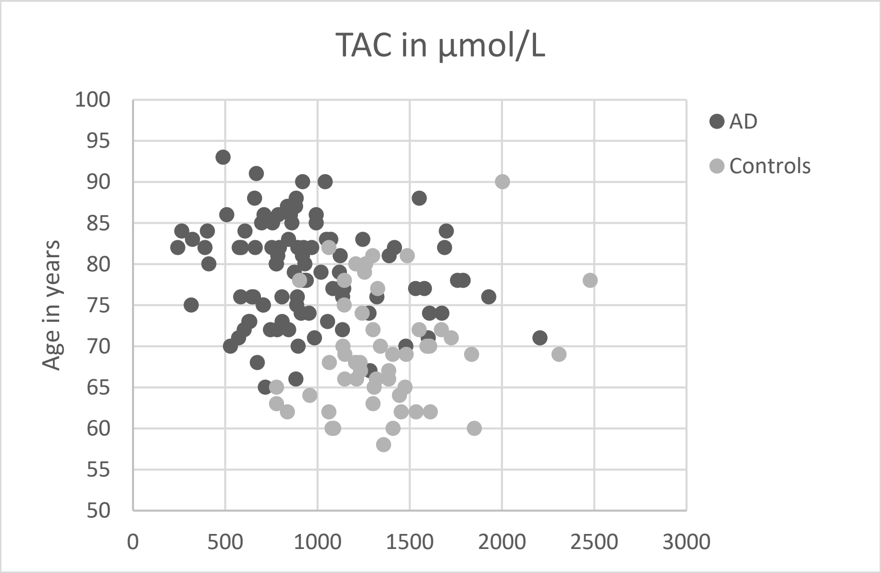

Supplementary Figure S1. Distribution of AOPP, fructosamine, and TAC levels by age group and disease status

Abbreviations: AOPP – advanced oxidation protein products, TAC – total antioxidant capacity, AD – Alzheimer's disease
